# Conservation of genetic diversity hotspots of the high‐valued relic yellowhorn (*Xanthoceras sorbifolium*) considering climate change predictions

**DOI:** 10.1002/ece3.4944

**Published:** 2019-03-01

**Authors:** Ren‐Bin Zhu, Qing Wang, Wen‐Bin Guan, Yanjia Mao, Bin Tian, Ji‐Min Cheng, Yousry A. El‐Kassaby

**Affiliations:** ^1^ Xishuangbanna Tropical Botanical Garden Chinese Academy of Sciences Menglun China; ^2^ College of Resource and Environment Northwest A&F University Yangling China; ^3^ School of Nature Conservation Beijing Forestry University Beijing China; ^4^ Department of Forest and Conservation Sciences, Faculty of Forestry The University of British Columbia Vancouver British Columbia Canada; ^5^ Key Laboratory of Biodiversity Conservation in Southwest China State Forestry Administration, Southwest Forestry University Kunming China; ^6^ Institute of Soil and Water Conservation Chinese Academy of Sciences and Ministry of Water resources Yangling China

**Keywords:** breeding, climate change, conservation, genetic diversity, *Xanthoceras sorbifolium*, yellowhorn

## Abstract

Genetic structure and major climate factors may contribute to the distribution of genetic diversity of a highly valued oil tree species *Xanthoceras sorbifolium* (yellowhorn). Long‐term over utilization along with climate change is affecting the viability of yellowhorn wild populations. To preserve the species known and unknown valuable gene pools, the identification of genetic diversity “hotspots” is a prerequisite for their consideration as in situ conservation high priority. Chloroplast DNA (cpDNA) diversity was high among 38 natural populations (*H*_d_ = 0.717, *K* = 4.616, Tajmas’ *D* = −0.22) and characterized by high genetic divergence (*F*_ST_ = 0.765) and relatively low gene flow (*N*_m_ = 0.03), indicating populations isolation reflecting the species’ habitat fragmentation and inbreeding depression. Six out of the studied 38 populations are defined as genetic diversity “hotspots.” The number and geographic direction of cpDNA mutation steps supported the species southwest to northeast migration history. Climatic factors such as extreme minimum temperature over 30 years indicated that the identified genetic “hotspots” are expected to experience 5°C temperature increase in next following 50 years. The results identified vulnerable genetic diversity “hotspots” and provided fundamental information for the species’ future conservation and breeding activities under the anticipated climate change. More specifically, the role of breeding as a component of a gene resource management strategy aimed at fulfilling both utilization and conservation goals.

## 1 INTRODUCTION

The International Union for Conservation of Nature and Natural Resources (IUCN) recognized the need for biodiversity conservation at its three levels: genetic, species, and ecosystem (McNeely, Miller, Reid, Mittermeier, & Werner, 1990). Conservation of genetic diversity is fundamental for species and ecosystem scales (Frankham, Ballou, & Briscoe, 2010). The importance of genetic diversity can be addressed as to reducing inbreeding depression (Frankham et al., 2010), providing fundamental resources for species evolution (Frankel & Bennett, 1970), enhancing population fitness, and decreasing extinction risk (Vellend & Geber, 2005). Although genetic diversity conservation was proposed as an explicit goal in 1993 (Burhenne‐Guilmin & Casey‐Lefkowitz, 1993), the conservation of genetic resources only focused on species of recognized value to humans. However, the value of vast genetic resources may still remain unknown and has been described as “sitting on the shelf” or “gene morgues” (Hoisington et al., 1999), such as those of yellowhorn (*Xanthoceras sorbifolium*).

**Figure 1:**
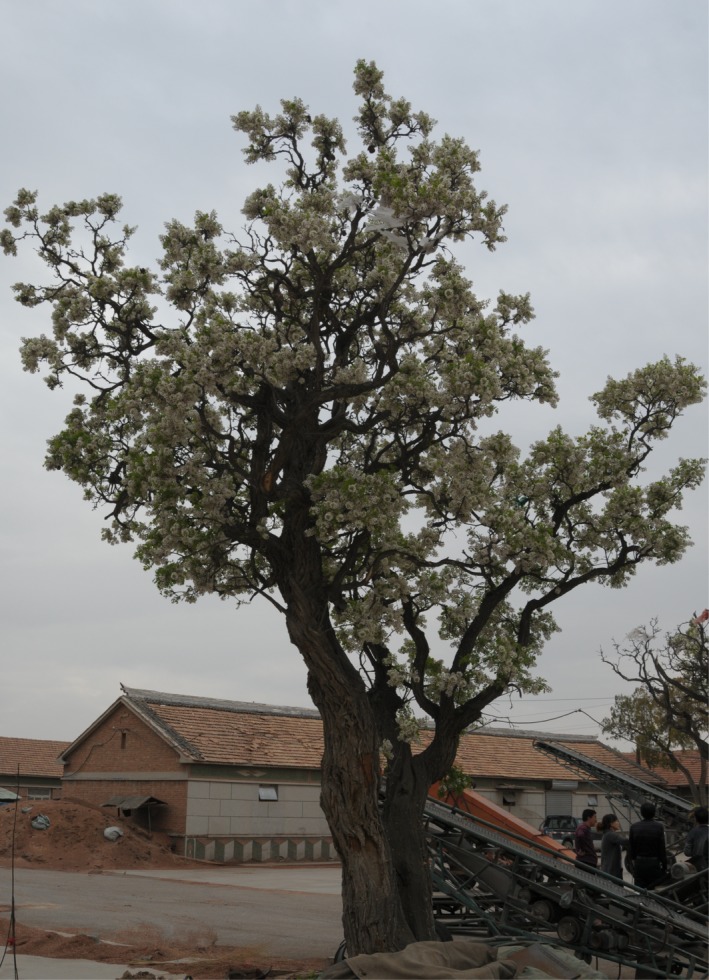
Yellowhorn tree in bloom. (photograph credit: Dr. Wen‐Bin Guan, School of Nature Conservation, Beijing Forestry University, Beijing, P.R. China)

Yellowhorn is a native relic‐species endemic to Northern China (Figure 1) (Fu et al., 2008; Wang, Huang, Wang, El‐Kassaby, & Guan, 2017; Wang, Yang et al., 2017). With its remarkable oil (Fu et al., 2008) and immeasurable medicinal value of seed and fruit shell (Chan & Mak, 2006; Ma, Nakamura, Nawawi, Hattori, & Cai, 2004; Zhang et al., 2010), yellowhorn has recently received increased research attention, including studies on fruit quality and genetic diversity (Wang, Huang et al., 2017; Wang, Yang et al., 2017); however, additional work is needed to explore the species entire natural range and the impact of climate change on its genetic diversity.

Climate change may affect the genetic diversity within and among populations and thus impacting species’ evolutionary potential (Vellend & Geber, 2005). The current observed rate of climate change is faster than that of postglacial warming (Willis & MacDonald, 2011) and is considered as a pervasive global problem (Hegerl et al., 2007). Thus, the reliance on long‐term evolutionary factors for creating new genes is not an option. To preserve yellowhorn's known and unknown valuable gene pools, the identification of the species genetic diversity “hotspots” is a prerequisite for their consideration as in situ conservation high priority (Shafer, Cullingham, Cote, & Coltman, 2010; Weiss & Ferrand, 2007). Additionally, predicting the magnitude of climate change and its impact on the genetic diversity within these “hotspots” is essential for the species future utilization and conservation strategies.

Breeding practices often focus on a subset of the base population, thus effectively reducing the genetic diversity (Chaisurisri & El‐Kassaby, 1994; El‐Kassaby & Ritland, 1996; Stoehr & El‐Kassaby, 1997); however, breeding activities should be viewed as a component of larger gene resource management strategy aimed at fulfilling both utilization and conservation goals. Wild genetic resources substantially contribute to conventional crop improvement efforts (e.g., wheats and maize (Hoisington et al., 1999)) and it should be noted that substantial numbers of the selected and deployed valuable varieties originated from landraces or nature individuals (Hoisington et al., 1999). Historically, tree selective breeding is a young practice started in 1950s, indicating that the state of forest tree genetic resources is still resembling their wild ancestors (White, Adams, & Neale, 2007). Most tree selective breeding efforts are mainly focused on economically valuable attributes with emphases on increasing yield and to a lesser extent quality. Exploring future tree genetic resources is anticipated to include additional attributes such as wood quality, drought, frost, and pest resistant or tolerance, and fruit yield and taste.

Chloroplast DNA (cpDNA) is haploid and nonrecombining genome (Comes & Kadereit, 1998), usually maternally inherited in most angiosperms such as Theaceae (Li, Awasthi, Yang, & Li, 2013) and some gymnosperms such as Ginkgo (Shen et al., 2004). cpDNA has been widely used in phylogeny, classification, and biogeography of many plant species (Olmstead & Palmer, 1997). In this study, we used cpDNA sequencing from five noncoding regions to assess haplotype diversity and relationship in 399 yellowhorn individuals representing 38 wild populations. We focused on three issues: (a) uncover the genetic diversity, genetic structure, and phylogenetic relationships; (b) identify the genetic diversity “hotspots” and conservation role under climate change; and (c) utility of breeding with valuable genotypes.

## 2 METHODS

### 2.1 Material collection

**Figure 2:**
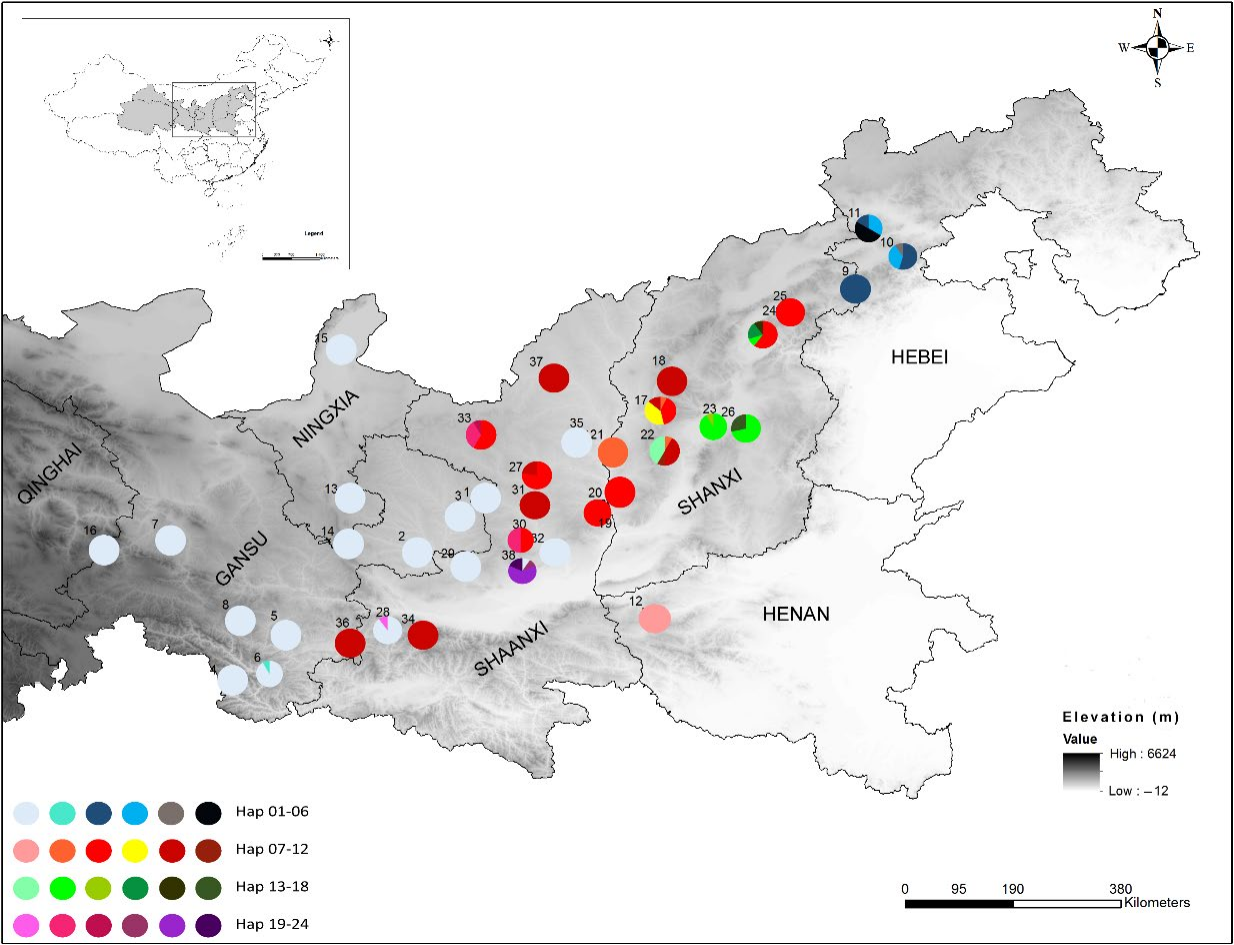
Geographical distribution of cpDNA haplotypes in 38 wild yellowhorn populations with their pie chart representations (colors are proportional representation of individual haplotype as illustrated in Table 1)

We sampled 399 individuals from 38 wild yellowhorn (*X. sorbifolium*) populations located in seven Chinese provinces (Gansu, Ningxia, Qinghai, Shaanxi, Shan'xi, Henan, and Hebei; Figure 2 and Table 1). Most of the sampled areas are located on the Loess Plateau, an arid or semi‐arid region, with 200–700 mm of annual precipitation, and occupy an elevational band between 800 and 2,200 m. From each individual tree, a fresh sample of 2–10 leaves was collected and stored in silica gel until further use. Due to the vegetative propagation nature of yellowhorn that often creates clonal clumps, sampled trees were intentionally separated by a minimum distance of 100 m (Song, Yin, Liu, Wang, & Jiao, 2011). All sampled locations were recorded using GPS HOLUX M‐241.

**Table 1:** Distribution of the cpDNA microsatellite haplotype and their estimates of diversity in 38 yellowhorn populations

Population	Provence	Longitude	Latitude	Elevation (m)	Haplotypes (no. of individuals)	Number	*H*_d_ (*SE*)	*K* (*SE*)	Tajima's *D*
1	Gansu	108°32′	36°10′	1,163	H01 (12)	12	0.000	0.000	nv
2	Gansu	107°29′	35°40′	1,120	H01 (09)	9	0.000	0.000	nv
3	Gansu	108°19′	36°04′	1,360	H01 (12)	12	0.000	0.000	nv
4	Gansu	104°31′	33°29′	1,443	H01 (12)	12	0.000	0.000	nv
5	Gansu	105°27′	34°14′	1,700	H01 (12)	12	0.000	0.000	nv
6	Gansu	105°01′	33°48′	1,400	H01 (11), H02 (01)	12	0.167	0.167	−1.141
7	Gansu	103°39′	35°59′	2,200	H01 (09)	9	0.000	0.000	nv
8	Gansu	104°50′	34°32′	2,000	H01 (09)	9	0.000	0.000	nv
9	Hebei	114°32′	39°59′	1,147	H03 (10)	10	0.000	0.000	nv
10	Hebei	115°03′	40°05′	1,186	H03 (06), H04 (04), H05 (01)	11	0.618	1.200	0.587
11	Hebei	114°51′	40°52′	1,062	H03 (02), H04 (03), H06 (05)	10	0.689	1.378	1.077
12	Henan	111°05′	34°38′	623	H07 (10)	10	0.000	0.000	nv
13	Ningxia	106°23′	36°15′	1,944	H01 (12)	12	0.000	0.000	nv
14	Ningxia	106°17′	35°48′	1,860	H01 (11)	11	0.000	0.000	nv
15	Ningxia	106°07′	38°53′	1,451	H01 (10)	10	0.000	0.000	nv
16	Qinghai	102°41′	35°49′	1,961	H01 (12)	12	0.000	0.000	nv
17	Shanxi	111°14′	37°55′	1,278	H08 (01), H09 (06), H10 (06), H11 (02)	15	0.705	1.981	−1.066
18	Shanxi	111°42′	38°18′	1,208	H11 (06)	6	0.000	0.000	nv
19	Shanxi	110°54′	36°13′	1,092	H09 (12)	12	0.000	0.000	nv
20	Shanxi	110°41′	36°09′	1,086	H09 (12)	12	0.000	0.000	nv
21	Shanxi	110°44′	37°01′	1,085	H08 (11)	11	0.000	0.000	nv
22	Shanxi	111°24′	37°01′	1,183	H08 (01), H11 (05), H12 (01), H13(05)	12	0.697	2.045	−1.573
23	Shanxi	112°23′	37°42′	973	H14 (05), H15 (01)	6	0.333	0.333	−0.933
24	Shanxi	113°17′	39°13′	1,026	H09 (06), H14 (01), H16 (02), H17 (01)	10	0.644	4.489	−0.426
25	Shanxi	113°34′	39°20′	1,294	H09 (11)	11	0.000	0.000	nv
26	Shanxi	112°52′	37°37′	814	H14 (05), H18(02)	7	0.476	4.286	0.886
27	Shaanxi	109°16′	36°50′	1,219	H11 (10)	10	0.000	0.000	nv
28	Shaanxi	107°05′	34°20′	802	H09 (07), H11 (03)	10	0.467	0.467	0.820
29	Shaanxi	108°08′	35°13′	1,163	H01 (09), H19 (01)	10	0.200	0.600	−1.562
30	Shaanxi	109°21′	35°59′	945	H01 (11)	11	0.000	0.000	nv
31	Shaanxi	109°20′	36°14′	1,060	H09 (05), H19 (05)	10	0.556	1.111	1.844
32	Shaanxi	109°50′	35°37′	1,168	H11 (10)	10	0.000	0.000	nv
33	Shaanxi	108°34′	37°27′	16,89	H01 (13)	13	0.000	0.000	nv
34	Shaanxi	107°45′	34°09′	771	H09 (07), H20 (04), H21 (01)	12	0.591	1.136	0.472
35	Shaanxi	110°12′	37°19′	1,000	H01 (09)	9	0.000	0.000	nv
36	Shaanxi	106°41′	34°03′	1,123	H11 (10)	10	0.000	0.000	nv
37	Shaanxi	109°48′	38°16′	1,084	H11 (08)	8	0.000	0.000	nv
38	Shaanxi	109°04′	35°10′	1,037	H01 (01), H22 (01), H23 (07), H24 (02)	11	0.600	0.691	−1.114
Mean							0.177 (0.04)	0.523 (0.18)	
Total					24	399	0.760	4.616	−0.220

Note

*H*_d_: haplotype diversity; *K*: average number of nucleotide differences; nv: no values for single haplotype.

### 2.2 DNA extraction, PCR amplification, and sequencing

Total genomic DNA was extracted from the leaf material following the cetyltrimethylammonium bromide (CTAB) procedure (Clarke, 2009). For PCR amplification, we used the following five pairs of universal cpDNA primers: *psbA*‐*trnH *(Harrington et al., 2005), *trnD*‐*trnT *and *trnS*‐*trnG* (Shaw et al., 2005), *rpl32*‐*trnL* (Shaw, Lickey, Schilling, & Small, 2007), and *trnL*‐*trnF* (Taberlet, Gielly, Pautou, & Bouvet, 1991). The amplification was carried out in 25 µl of reaction mixture, containing 9.5 µl dd H_2_O, 12.5 µl Mix, 2 µl primer (5′ and 3′ ends), and 1 µl of genomic DNA. The amplification for all the chloroplast regions consisted of 4 min at 94°C, followed by 3 cycles of (30 s at 94°C, 45 s at 55°C, and 1 min at 72°C), 3 cycles of (30 s at 94°C, 45 s at 53°C, and 1 min at 72°C), 3 cycles of (30 s at 94°C, 45 s at 54°C, and 1 min at 72°C), 3 cycles of (30 s at 94°C, 45 s at 56°C, and 1 min at 72°C), 23 cycles of (30 s at 94°C, 45 s at 58°C, and 1 min at 72°C), extending with 10 min at 72°C, finishing with 4°C. PCR products detected using polyacrylamide gel electrophoresis (PAGE) at 120 V for 5 hr (agarose gel. (0.8 g) for run PCR products in 1X TAE along with 1 kb ladder as molecular size marker; 0.5 µl 4S Red Plus Acid Stain for visualization). Sequencing was performed at Sangon Biotech (Shanghai, China). All the sequences were manually checked and edited using MEGA 6.0 (Tamura, Stecher, Peterson, Filipski, & Kumar, 2013). Length for the internal transcribed spacers (ITSs) was 517, 1,144, 1,650, 963, and 571 bp for *psbA*‐*trnH*, *trnD*‐*trnT*, *rpl32*‐*trnL*, *trnL*‐*trnF*, and *trnS*‐*trnG*, respectively, generating a sequence of 4,575 bp allowing detecting 32 intergenic spacer (IGS) regions with identified 24 haplotypes.

### 2.3 Data analysis

#### 2.3.1 Analysis of genetic structure and diversity

Haplotype diversity (*H*_d_; (Hudson, Boos, & Kaplan, 1992)) and average number of nucleotide differences (*K*; (Nei, 1973)) were calculated using the function “get.diversity” and “Pi,” and Tajima's *D* (Tajima, 1989) for selective neutrality or population size changes by using “Tajima.D” function. Haplotype divergence *F*_ST_ (Hudson et al., 1992) was calculated by “get.F_ST” function in the “popgenome” package (Pfeifer, Wittelsbürger, Ramos‐Onsins, & Lercher, 2014). Genetic abundance distribution and cluster for the 38 yellowhorn populations were conducted using the “heatmap” function in the “gplots” package (Warnes et al., [Link]). Haplotype network and frequency were determined using “haploNet” function in the “pegas” package (Paradis, 2010). All analyses were conducted in R version 3.3.1 (R Development Core Team, 2010).

#### 2.3.2 Phylogenetic relationships and analyses

Phylogenetic relationships based on cpDNA sequence data were conducted using the Bayesian phylogenetic analysis following the online version of the program MrBayes at CIPRES Portal v.3.3 (Miller, Pfeiffer, & Schwartz, 2010) with *Aesculus assanuca*, *Acer coriaceifolium*, and *Koelreuteria paniculata* as out‐group. PAUP and RAxML‐HPC BlackBox Toolbox in CIPRES Portal v.3.3 (Miller et al., 2010) contributed to the built phylogenetic tree base on maximum parsimony (MP) and maximum likelihood (ML), respectively. The phylogenetic tree based on Bayes, ML, and MP was constructed using FigTree v 1.4.3 (available at http://tree.bio.ed.ac.uk/software/figtree/).

#### 2.3.3 Identifying climate variables associated with individual‐based genetic diversity

We used canonical correspondence analysis (CCA) ordination to identify the climate variables that are correlated with the distribution of haplotype diversity. CCA was calculated by the “cca” function in package “vegan” in R 3.3.1 (Oksanen et al., 2007), and it is based on chi‐squared distances and performs weighted linear mapping. In CCA, “species matrix” is the haplotype abundance in populations. A total of 16 climate variables (MAT: mean annual temperature (°C); MWMT: mean warmest month temperature (°C); MCMT: mean coldest month temperature (°C); TD: temperature difference between MWMT and MCMT, or continentality (°C); MAP: mean annual precipitation (mm); MSP: mean summer (May–September) precipitation (mm); AHM: annual heat moisture index (MAT + 10)/(MAP/1,000); DD < 0: degree‐days below 0°C, chilling degree‐days; DD > 5: degree‐days above 5°C, growing degree‐days; DD < 18: degree‐days below 18°C, heating degree‐days; DD > 18: degree‐days above 18°C, cooling degree‐days; NFFD: the number of frost‐free days; PAS: precipitation as snow (mm) between August in previous year and July in current year; EMT: extreme minimum temperature over 30 years; Eref: Hargreaves reference evaporation; CMD: Hargreaves climatic moisture deficit) were determined for present, 2050, and 2070 for each population using the software package Climate AP (Wang, Wang, Innes, Seely, & Chen, 2018).

## 3 RESULTS

### 3.1 Genetic structure and genetic diversity “hotspots” identification

**Figure 3:**
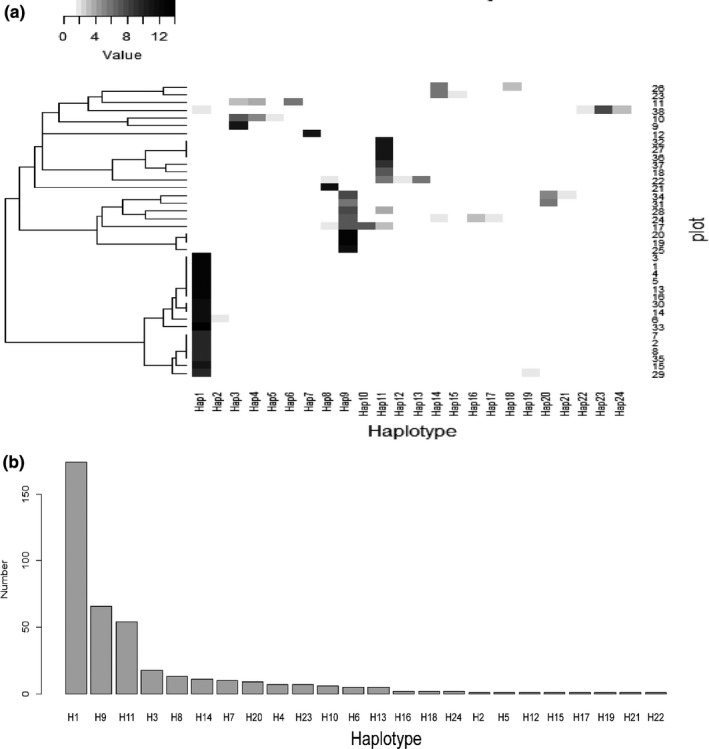
(a) “Heatmap” representing the correlations among the 38 populations and 24 haplotypes; (b) ranking based on the number of individuals per haplotype

A total of 24 haplotypes were found in the 399 individuals representing the 38 wild yellowhorn populations with 21 populations producing only one single haplotype (Figure 2; Table 1). Among the 38 populations, three haplotypes (H1 (174/399), H9 (66/399), and H11 (54/399)) were widely distributed and accounted for 74% and 82% of the total investigated individuals and populations, respectively (Figure 3 a and b). The yellowhorn maintained high genetic diversity across the 38 populations (haplotype diversity (*H*_d_) = 0.760; average number of nucleotide differences (*K*) = 4.616, with six populations (#10, 11, 17, 22, 24, and 38) harboring high haplotype diversity (*H*_d_: ranged between 0.600 and 0.705) and could be recognized as genetic “hotspots” (Table 1). The mean *F*_ST_ (population subdivision) and *N*_m_ (gene flow) were 0.893 and 0.03, respectively, indicating that the observed genetic difference mainly occurred among rather than within populations. Tajima's *D* neutrality test varied among the 38 populations but did not differ significantly from zero (Table 1).

### 3.2 Phylogenetic relationships and cluster among wild populations

**Figure 4:**
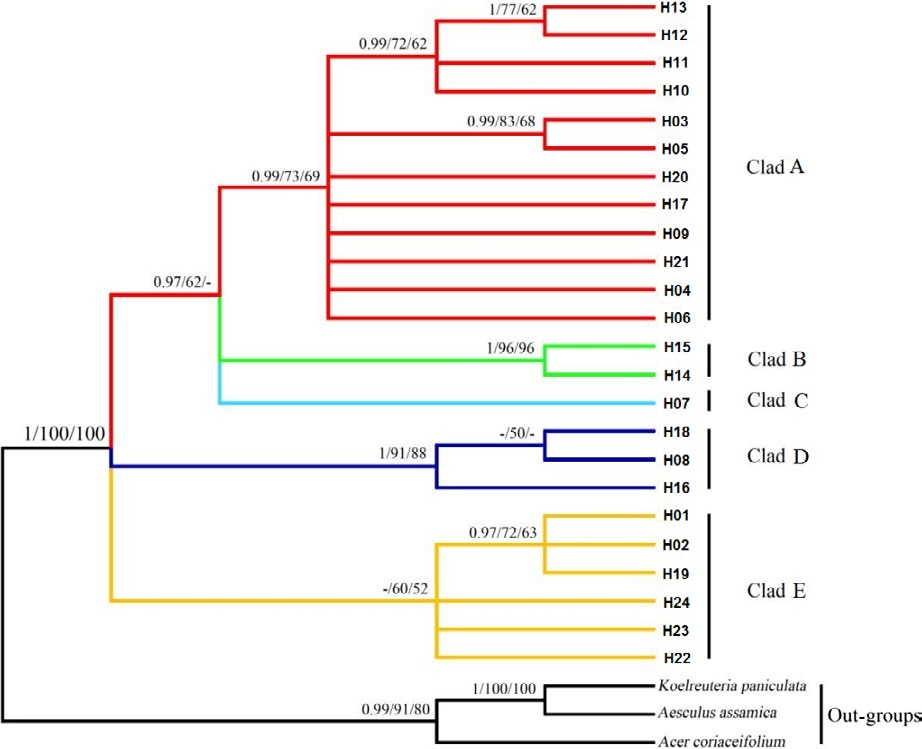
Phylogenetic tree obtained from posterior probabilities (>0.9) based on Bayes and bootstrap values (>50%), maximum‐likelihood (ML), and maximum parsimony (MP) analyses

**Figure 5:**
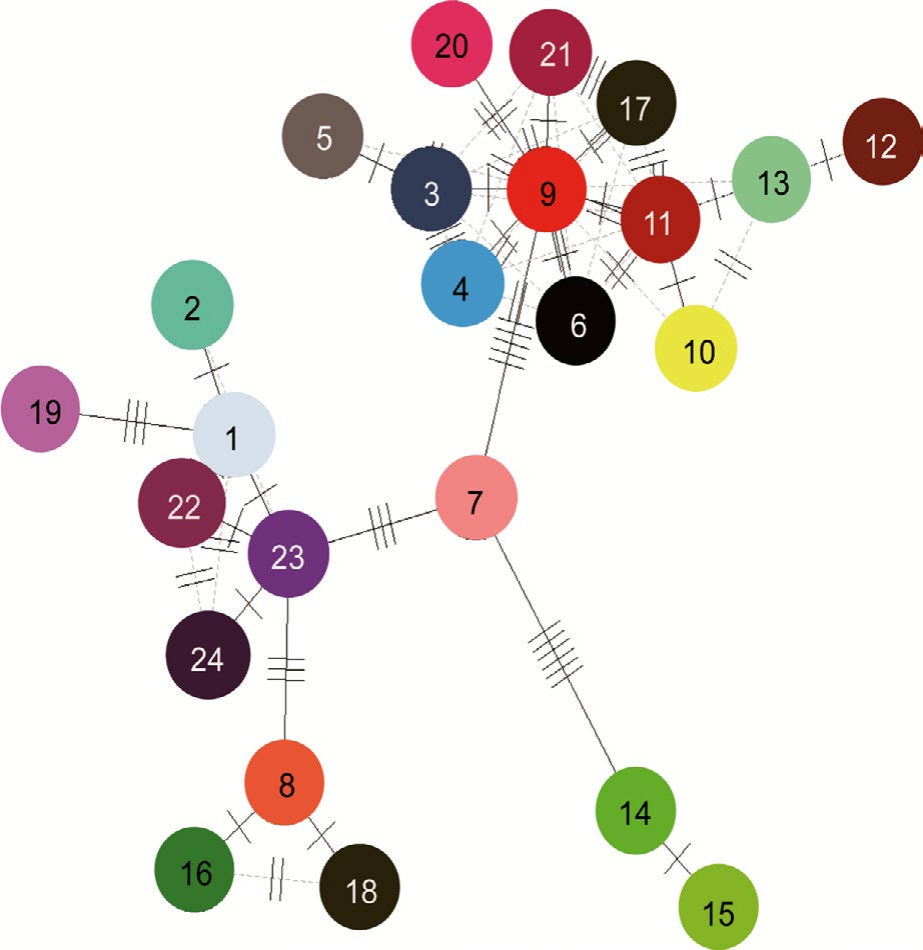
Network for 24 cpDNA microsatellite haplotypes (solid bars indicate the number of mutational steps. See Figure 1 for haplotype colors)

Based on Bayes (posterior probabilities >0.9), maximum parsimony (MP), and maximum likelihood (ML) (bootstrap value >50%), the phylogenetic relationships among the 24 yellowhorn cpDNA microsatellite haplotypes showed similar results (Figure 4). Five major clusters (clade A, B, C, D, and E) were observed along with *Koelreuteria paniculata*, *Aesculus assamica*, and *Acer coriaceifolium* as out‐groups. Clade A (H3, H4, H5, H6, H9, H10, H11, H12, H13, H17, H20, and H21) is mainly distributed in the northern part of Luliang (Shanxi Province) and Taihang (mainly in Hebei and partially in Henan and Shanxi Provinces) Mountains, B (H14 and H15) is representative of the Taiyuan Basin (Taiyuan Province), C (H7) located in northeastern Qinling Mountain (Shaanxi Province) and east of the Yellow River, D (H8, H16, and H18) representing the western part of Luliang Mountain (Shanxi Province), and E (H1, H2, H19, H22, H23, and H24) distributed in the western part of Ziwuling Mountain (Shaanxi and Gansu Provinces). The 24 cpDNA microsatellite haplotypes network analysis indicated that there are 6 mutation steps between H7 and H14, 5 steps between H7 and H9, and 3 steps between each of H7 and H23, H8 and H23, and H1 and H19 (Figure 5). Haplotype H1 is commune in the southwestern region while haplotype diversity is increasing from southwest to northeast (Figures 2 and 4).

### 3.3 Influence of climate factors on yellowhorn cpDNA microsatellite haplotype distribution

**Figure 6:**
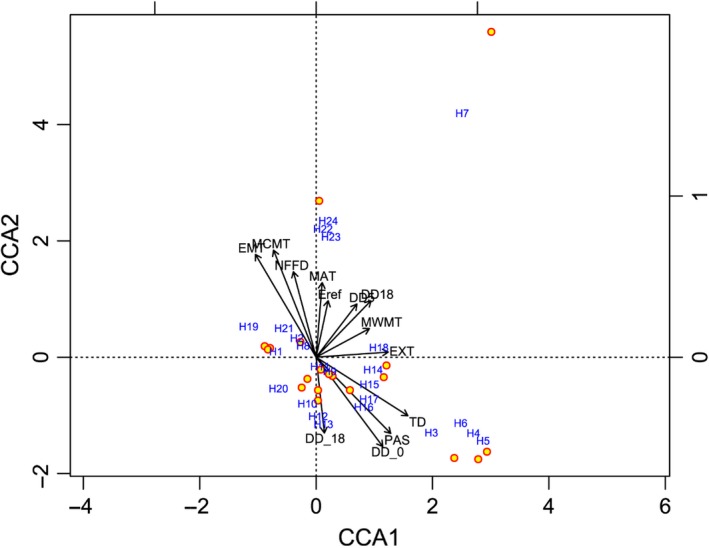
Canonical correspondence analysis (CCA) for cpDNA microsatellite haplotypes diversity in 38 yellowhorn populations’ fit with climate variables (see Table 2 for climate factors’ abbreviations). Yellow dots and blue numbers represent 38 and 24 wild populations and haplotypes, respectively. Arrows represent climate factors and their length is proportionate to the correlation level

**Figure 7:**
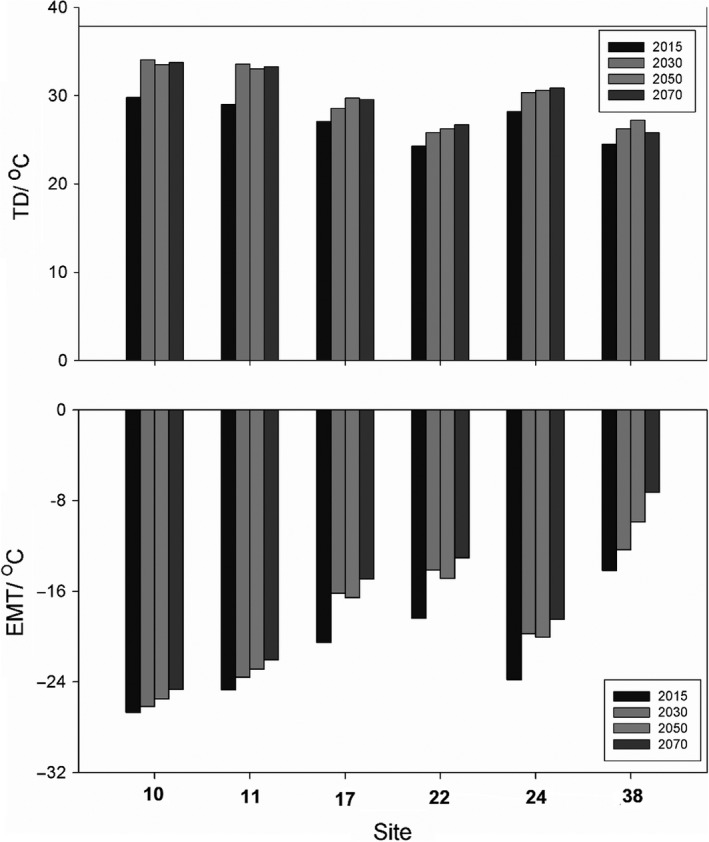
Significant climate variables changing in recognized genetic diversity “hotspots” for next 50 years

Based on the canonical correspondence analysis (CCA) ordination results, 12 (mean annual temperature (MAT), mean warmest month temperature (MWMT), mean coldest month temperature (MCMT), temperature difference between MWMT and MCMT (TD), degree‐days below 0°C (DD < 0), degree‐days above 5°C (DD5), degree‐days above 18°C (DD < 18), number of frost‐free days (NFFD), precipitation as snow (PAS), extreme minimum temperature over 30 years (EMT), extreme maximum temperature over 30 years (EXT), and Hargreaves reference evaporation (Eref)) out of the 16 studied climate factors significantly associated with haplotype distribution (Figure 6; Table 2). MAT, MWMT, MCMT, DD5, DD18, NFFD, EMT, EXT, and Eref aggregated on the positive side of the CCA1 axis, while TD, DD < 0, DD < 18, and PAS aggregated on the negative side (Figure 6). Among the studied climate factors, MCMT, TD, DD < 0, and EMT showed highly significant positive (or negative) association with the cpDNA microsatellite haplotypes distribution. Both TD and EMT in the identified haplotype diversity “hotspots” (populations with high haplotype diversity, including populations 10, 11, 17, 22, 24, 38) will increase by 3 to 5°C in the following 50 years (Figure 7).

**Table 2:** Correlations of climate variables with the first (CA1) and second (CA2) canonical correspondence analysis axes and yellowhorn cpDNA microsatellite haplotypes (*n* = 399)

Climate factor	CA1	CA2	*r* ^2^	*p*
MAT	0.93345	0.35872	0.2262	0.011*
MWMT	0.36903	0.92942	0.1722	0.033*
MCMT	0.98565	−0.16878	0.4147	0.001***
TD	−0.59951	0.80037	0.3788	0.001***
DD < 0	−0.97128	0.23795	0.4283	0.001***
DD5	0.66178	0.7497	0.1825	0.027*
DD < 18	−0.9753	−0.22087	0.2341	0.009**
DD18	0.59607	0.80293	0.2026	0.011*
NFFD	0.99955	−0.03001	0.2386	0.010**
PAS	−0.91302	0.4079	0.3824	0.002**
EMT	0.91628	−0.40055	0.4086	0.001***
EXT	0.02155	0.99977	0.2033	0.019*
Eref	0.8274	0.56161	0.2395	0.007**

Note

DD < 0: degree‐days below 0°C, chilling degree‐days; DD < 18: degree‐days below 18°C, heating degree‐days; DD > 18: degree‐days above 18°C, cooling degree‐days; DD > 5: degree‐days above 5°C, growing degree‐days; EMT: extreme minimum temperature over 30 years; Eref: Hargreaves reference evaporation; EXT: extreme maximum temperature over 30 years; MAT: mean annual temperature (°C); MCMT: mean coldest month temperature (°C); MWMT: mean warmest month temperature (°C); NFFD: the number of frost‐free days; PAS: precipitation as snow (mm) between August in previous year and July in current year; *r* ^2^: Coefficient of determination; TD: temperature difference between MWMT and MCMT, or continentality (°C).

*** *p* < 0.001,

** *p* < 0.01,

* *p* < 0.05.

## 4 DISCUSSION

### 4.1 Genetic structure genetic diversity and phylogenetic relationships

In the present analyses, wild yellowhorn populations maintained high haplotype diversity (*H*_d_ = 0.760, *K* = 4.616) compared to other species (average haplotype diversity for 170 species is 0.67 (Petit et al., 2005)) and high genetic difference (*F*_ST_ = 0.893), but very low gene flow (*N*_m_ = 0.03) among the studied 38 populations, while no individual population value of Tajima's D was significantly different from random expectations. Similar results also found in other long‐lived tree species (e.g., *Alsophila spinulosa*: *F*_ST_ = 0.92, *N*_m_ = 0.09 (Su et al., 2005); *Taxus wallichiana*: *F*_ST_ = 0.884 with low levels of recurrent gene flow (Gao et al., 2007)). This may be associated with the long evolutionary history for woody species. Genetic diversity is controlled by four processes, including mutation, drift, migration, and selection (Hoisington et al., 1999). Tajima's D is widely used to evaluate the pattern of demographic processes within species (genetic bottlenecks (or genetic drift) and founder effects), which may cause population structure and selection (Mahoney, 2004; Ptak & Przeworski, 2002). Negative and positive values of within populations Tajima's *D* indicate that populations have undergone demographic expansions and experienced bottlenecks, respectively (Tajima, 1989). According to our results, populations 6, 17, 22, 23, 24, 29, and 38 may have undergone demographic expansions under historical climate change, while populations 10, 11, 26, 28, 31, and 34 may have experienced bottlenecks during their evolutionary journey.

Fruit of yellowhorn form capsules, those capsules disclose when ripe (July–August), the capsule shells become hard and woody. These fruits contain on average 15–25 seeds (with hard seed coats) (Zhou & Liu, 2012), and mean single seed weight is around 0.87 g (Wang, Huang et al., 2017; Wang, Yang et al., 2017). As in most plant species, relatively heavy seed is dispersed within short distance (dozen meters) (Willson, 1993). The evolution of plants is affected by long‐distance dispersal (LDD). LDD is influenced by several factors including the presence of open terrestrial landscapes and mediation by large animals, migratory animals, extreme meteorological events, ocean currents, and human transportation (Nathan et al., 2008) as well as extreme phenomenon such as glaciation and continental drift (Smith, 1993).

Yellowhorn is an ancient woody perennial species belonging to Xanthoceroideae family that evolved during the Late Cretaceous (110 My) with other species in Sapindaceae (including four lineages: Xanthoceroideae, Hippocastanoideae, Dodonaeoideae, and Sapindoideae; Buerki et al., 2011). Yellowhorn original distribution is the tropics (like other species in Sapindaceae, eg. Longan (Nakasone & Paull, 1998)), then migrated north to temperate region. In our study, H1 is the most widely distributed haplotypes (Figure 3) and may be dispersed in the relatively flat landscape by animals. In fact, the Siberian chipmunk (*Eutamias sibiricus*) that was frequently observed in the studied populations could have acted as a vector aiding the spreading of this haplotype. The presence of the Ziwuling Mountain that occurred in 1.67–1.45 Ma BP (Zhaoyu, 1992) may have contributed to separating clades E (western part of Ziwuling Mountain) and A; Taiyuan Basin formed around 7.2–5.3 Ma (Ke, 2012), surrounded by Luliang and Taihang Mountains and Fenhe River (in Shanxi Province), could have contributed to the isolation of clade B from other clades; clade D representing the western part of Luliang Mountain which is surrounded by the Fenhe River could be responsible for the species’ observed contemporary distribution. Finally, the isolated evolution of clade C which is distributed in northeastern Qinling Mountain may have separated it from other clades by the occurrence of the Yellow River in 0.15 Ma (Zheng et al., 2007). Population's genetic diversity showed substantial reduction from northeast to southwest regions (Figures 2 and 4) and may be due to the isolation by mountains and rivers forcing yellowhorn to evolve to adapt to the new environments during its northeastward migration. According to the tropical origin hypothesis, H1 is the most ancient haplotype, the pattern of mutation steps highly support the northeastward migration route (Figure 5).

### 4.2 Genetic diversity conservation under climate change

In the present time, the rate and magnitude of warming may be comparable to that of postglacial warming (Solomon, 2007; Willis & MacDonald, 2011). Climate warming can reduce genetic richness by indirectly reducing landscape and species diversity (Chapin III et al., 2000). Understanding among and within genetic diversity indices will help identifying the genetic diversity “hotspots” and unique haplotypes. Moreover, identifying climate factors which contribute to the distribution of genetic diversity may provide ideas for genetic conservation under climate change. In light of the present study, genetic “hotspot” conservation is the most efficient way to protect the yellowhorn's genetic diversity (i.e., the identified six wild yellowhorn populations harboring higher haplotype diversity). Climate variables TD (temperature difference between MWMT (mean warmest month temperature) and MCMT (mean coldest month temperature)) and EMT (extreme minimum temperature over 30 years) in the identified haplotype diversity “hotspots” are expected to increase by 3–5°C in the following 50 years (Figure 7). TD showed the difference between mean warmest month temperature (MWMT) and mean coldest month temperature (MCMT) is increasing, while the extreme minimum temperature over 30 years (EMT) is increasing, which indicate that extreme weather conditions may occur more than contemporary observed. While yellowhorn is adapted to warm climate as its original distribution is in tropical region; however, the observed and predicted climate change are expected to cause greater challenges. Surprisingly, all moisture‐related climatic variables were nonsignificant indicating that temperature alone plays a major role in the species contemporary and future distributions and confirming the species drought resistance (An et al., 2016).

The cpDNA genome is characterized by its slow evolution caused by low mutation rate (Palmer & Herbon, 1988). Thus, the observed high haplotype diversity (*F*_ST_ > 0.2) that is coupled with very low gene flow indicates that yellowhorn populations have been isolated for a very long time and may undergo further habitat fragmentation. Furthermore, while the mode of inheritance of yellowhorn's chloroplast is unknown, it is expect to be material as most angiosperms (Reboud & Zeyl, 1994), thus limiting gene flow due to the species large seed (Wang, Huang et al., 2017; Wang, Yang et al., 2017), further contributing to fragmentation.

Wild yellowhorn often found in Versant Soleil occupying forest edges presumably for sunlight accessibility (personal field observation). This habitat distribution type is more sensitive to edge effects and habitat fragmentation which may be exasperated by global warming (Reinmann & Hutyra, 2017), and consequently contributing to woody plant ecosystem's properties and thus changing the habitat of animals associated with long‐distance dispersal (LDD). Thus, the migration and evolution of yellowhorn are expected to face natural and anthropogenic biogeographic barriers (Lumibao, Hoban, & McLachlan, 2017).

To achieve Aichi Biodiversity Targets (Goal A) in Convention on Biological Diversity (https://www.cbd.int/sp/targets/), the Chinese National Natural Conservation Areas program is providing the most restrictive laws to protect wild plant and animal species (Wang, Huang et al., 2017; Wang, Yang et al., 2017), and this prompted the establishment of the Sanjiangyuan National Park in 2017, the first National Park in China. According to condition 2 of the regulations of the People's Republic of China on Nature Reserves (2017 version: http://www.forestry.gov.cn/main/3950/content-459882.html), the natural distribution areas of rare or endangered species are considered as locations for the establishment of new protect area (national parks, nature reserves, and wilderness areas). Our yellowhorn previous research showed that the total distribution area of wild yellowhorn is expected to decline by 10%–17% (≈210,000–330,000 km^2^) in the next 30–50 years by the impact of global warming and identified the species as endangered in its wild populations (Wang, Huang et al., 2017; Wang, Yang et al., 2017). Thus, we suggest the establishment of yellowhorn protected areas in the identified six genetic diversity “hotspots,” and this initiative could help conserving the existing genetic diversity within these unique gene pools (Jenkins & Joppa, 2009; Maxted, Dulloo, Ford‐Lloyd, Iriondo, & Jarvis, 2008). Additionally, maintaining and conserving the yellowhorn ecosystem will aid in the conservation and protection of those animals associated with the species seeds dispersal. Furthermore, while efforts are dedicated to the in situ conservation of yellowhorn in its wild estate within protected ecosystems, a complementary ex situ conservation efforts such as gene banks are also advocated (Cohen, Williams, Plucknett, & Shands, 1991; Vavilov, 1927).

### 4.3 Breeding program based on utilization of genetic resources

Mountains and plateaus account to 70% of land area of China (Baiping, Shenguo, Ya, Fei, & Hongzhi, 2004). Considering the habitat of yellowhorn (tolerance to high pH, clay, sandy, loam, average, medium or well‐drained soil (Li et al., 2010)), there is large potential for planting yellowhorn. The main advantage of planting yellowhorn is the species ability to grow on marginal sites, thus does not compete on fertile land distant for other crops creating an economic and environmental “win–win” scenario (Wang, Huang et al., 2017; Wang, Yang et al., 2017). To enhance to development of yellowhorn planting, the Chinese government sponsored the “11th Five‐Year Plan” to ensure planting of more than 105 ha per year until 2020 (Yu et al., 2017). The present challenge is the identification of the best yellowhorn “varieties,” thus embarking on a breeding and selection program is expected to address this issue.

In reality, the selection and breeding of yellowhorn have started in 1970s, with traditional breeding methods following the recurrent selection scheme with its selection, breeding, and testing cycles. Presently, with the availability of affordable genomic markers and advanced computational methods, a more efficient breeding methods such as genomic selection (GS) could be implemented (Meuwissen, Hayes, & Goddard, 2013). This approach is expected to reduce traditional tree breeding methods’ protracted timelines and their dependence on sustained financial and administrative commitments (El‐Dien et al., 2016; El‐Kassaby, Funda, & Liewlaksaneeyanawin, 2015; El‐Kassaby & Klapště, 2015; Ratcliffe et al., 2015). It should be highlighted that the peculiar reproductive nature of yellowhorn (known as the species with “thousand flowers but one fruit” (Ding & Ao, 2008)) and alternative deployment methods through vegetative propagation are required to overcome the reproductive approach drawbacks (El‐Kassaby & Klapště, 2015). We feel that the adoption of genomic selection for identifying superior individuals and the implementation of vegetative propagation scheme (e.g., root cutting (Yao, Qi, & Yin, 2013)) will lead to a faster yellowhorn population development.

The advantage of genomic selection is its utility to domestic as well as wild populations. Conducting GS in wild populations offers opportunities and challenges. Opportunities include capitalizing on historical linkage disequilibrium between target traits and SNP (single nucleotide polymorphism; Neale & Savolainen, 2004), high selection intensity (many individuals to select from), utilizing natural mating thus bypassing conventional breeding, availability of genetic analyses to estimate traits heritability after pedigree reconstruction (El‐Kassaby & Lstibůrek, 2009), and extensive phenotypic variability, collectively all expect to advance the genetic gain for the target traits. On the other hand, the main challenges are age and environmental heterogeneity differences and these could be dealt with tree aging and statistical methods that account for spatial heterogeneity (Cappa et al., 2017). Previous research has shown that yellowhorn oil contains unsaturated very long chain fatty acids (VLCFAs: oleic, linoleic, gondoic, erucic, and nervoic acid (Zhang et al., 2010)) which is been widely used as edible oil, and in cosmetics, medicine, and biofuel (Venegas‐Calerón, Ruíz‐Méndez, Martínez‐Force, Garcés, & Salas, 2017). Thus, these attributes will form the foundation for phenotyping of economically valuable attributes during the implementation of GS. To maximize the chances for success, we propose implementing GS on the six identified “hotspots” wild yellowhorn populations.

Finally, it should be emphasized that breeding efforts are complementary to conservation, so it is expected that the majority of the unknown valuable attributes of yellowhorn will be safeguarded with this initiative.

## 5 CONCLUSION

The genetic structure and diversity of 38 yellowhorn wild populations were assessed using 399 individuals representing 7 provinces covering the species’ natural range. We use cpDNA microsatellite haplotype variation to determine the species contemporary variation and its population differentiation as affected by postglacial migration and the anticipated global warming. Six genetic diversity “hotspots” were identified and deemed important for high conservation priority. A utilization (breeding) and conservation initiative is proposed.

## CONFLICT OF INTEREST

None declared.

## AUTHORS’ CONTRIBUTION

Y.A.E. and J.C. conceived and designed the experiments. R.Z., Y.M, B.T., and J.C. performed the experiment. R.Z., Y.M, B.T., and Q.W. contributed to data analysis. R.Z., Q.W., W.G, and Y.A.E. wrote, edited, and reviewed the MS.

## Data Availability

Not applicable.
